# Neurobiological mechanisms and related clinical treatment of addiction: a review

**DOI:** 10.1093/psyrad/kkac021

**Published:** 2022-12-16

**Authors:** Yehong Fang, Yunkai Sun, Yi Liu, Tieqiao Liu, Wei Hao, Yanhui Liao

**Affiliations:** Department of Psychiatry, Sir Run Run Shaw Hospital, Zhejiang University School of Medicine, 3 East Qingchun Road, Hangzhou, Zhejiang 310016, China; Department of Psychiatry, Sir Run Run Shaw Hospital, Zhejiang University School of Medicine, 3 East Qingchun Road, Hangzhou, Zhejiang 310016, China; Department of Psychiatry, Sir Run Run Shaw Hospital, Zhejiang University School of Medicine, 3 East Qingchun Road, Hangzhou, Zhejiang 310016, China; Department of Psychiatry & Mental Health Institute of the Second Xiangya Hospital, Central South University. National Clinical Research Center on Mental Disorders & National Technology Institute on Mental Disorders. Hunan Key Laboratory of Psychiatry and Mental Health, 139 Renmin (M) Rd, Changsha, Hunan 410011, P. R. China; Department of Psychiatry & Mental Health Institute of the Second Xiangya Hospital, Central South University. National Clinical Research Center on Mental Disorders & National Technology Institute on Mental Disorders. Hunan Key Laboratory of Psychiatry and Mental Health, 139 Renmin (M) Rd, Changsha, Hunan 410011, P. R. China; Department of Psychiatry, Sir Run Run Shaw Hospital, Zhejiang University School of Medicine, 3 East Qingchun Road, Hangzhou, Zhejiang 310016, China

**Keywords:** addiction, substance use disorder, magnetic resonance imaging, neurobiological mechanisms, craving, treatment

## Abstract

Drug addiction or substance use disorder (SUD), has been conceptualized as a three-stage (i.e. binge/intoxication, withdrawal/negative affect, and preoccupation/anticipation/craving) recurring cycle that involves complex changes in neuroplasticity, reward, motivation, desire, stress, memory, and cognitive control, and other related brain regions and brain circuits. Neuroimaging approaches, including magnetic resonance imaging, have been key to mapping neurobiological changes correlated to complex brain regions of SUD. In this review, we highlight the neurobiological mechanisms of these three stages of addiction. The abnormal activity of the ventral tegmental, nucleus accumbens, and caudate nucleus in the binge/intoxication stage involve the reward circuit of the midbrain limbic system. The changes in the orbitofrontal cortex, dorsolateral prefrontal cortex, amygdala, and hypothalamus emotional system in the withdrawal/negative affect stage involve increases in negative emotional states, dysphoric-like effects, and stress-like responses. The dysregulation of the insula and prefrontal lobes is associated with craving in the anticipation stage. Then, we review the present treatments of SUD based on these neuroimaging findings. Finally, we conclude that SUD is a chronically relapsing disorder with complex neurobiological mechanisms and multimodal stages, of which the craving stage with high relapse rate may be the key element in treatment efficacy of SUD. Precise interventions targeting different stages of SUD and characteristics of individuals might serve as a potential therapeutic strategy for SUD.

## Introduction

Substance use disorder (SUD), also known as drug addiction, is a state of compulsive drug use despite substantial harm and adverse consequences (Franklin, 1995; Nathan *et al*., 2016; Volkow and Boyle, 2018). Generally, SUDs include alcohol use disorder, tobacco use disorder, and other illicit drug-use disorder (such as methamphetamine, ketamine, and heroin) (Petry *et al*., 2018). SUDs are characterized by (i) compulsion to seek and take the drug, (ii) loss of control in limiting intake, and (iii) emergence of a negative emotional state (e.g. dysphoria, anxiety, irritability) reflecting a motivational withdrawal syndrome when access to the drug is prevented (Koob and Volkow, 2016). According to George F. Koob and Nora D. Volkow's conceptual framework (Koob and Volkow, 2010), SUD can be summarized as a composite cycle composed of the three stages: binge/intoxication, withdrawal/negative affect, and preoccupation/anticipation (craving). The intoxicating and incentive salience, rewarding effects of drug use, and pathological habits (e.g. drug-seeking habits) are dysregulation of function in the binge/intoxication stage. Negative reinforcement mainly driven by negative emotion is a common presentation in the withdrawal/negative affect stage. In the preoccupation/anticipation (craving) stage, abnormal executive function results in a decline in cognitive control, which is a key element in relapse (reviewed in Koob and Volkow, 2016).

The neuroimaging findings in human and animals indicate that the binge/intoxication stage of drug addiction involves changes in dopamine (DA) and opioid peptides as well as other neurotransmitters in basal ganglia (Belin *et al*., 2009). Once addicted, the brain mesolimbic and mesocortical DA levels, crucial for reinforcing effects, are increased (Hyman *et al*., 2006). The neurons in the ventral tegmental area (VTA), both DA-releasing and non-DA-releasing projecting neurons, play a central role in rewarding circuitry and related drug seeking behaviour (Morales and Margolis, 2017). In parallel, the withdrawal stage mainly involves changes in the orbitofrontal lobe, dorsolateral prefrontal cortex (DLPFC) (Parvaz *et al*., 2011) and extended amygdala (EA) (Carmack *et al*., 2019; Volkow *et al*., 2019) related to withdrawal symptoms such as negative emotion states and stress. The preoccupation/anticipation stage involves changes in prefrontal cortex (PFC) and insula related to craving (Parvaz *et al*., 2011) and changes in dorsolateral prefrontal and cingulate gyrus related to disrupted inhibitory control (Goldstein and Volkow, 2002).

Regarding the current clinical therapy for SUD, this mainly includes pharmacological and nonpharmacological interventions (Volkow and Boyle, 2018; Kalin, 2020). To date, the Food and Drug Administration (FDA) has approved several medications for the treatment of different types of substance use disorder, such as buprenorphine/naloxone and methadone for opioid use disorder, acamprosate for alcohol use disorder, nicotine replacement therapies, bupropion, and varenicline for tobacco use disorder. However, some medicines are addictive with large side-effects, and most of them show unsatisfactory long-term efficacy (Volkow and Boyle, 2018). Similarly, psychological therapies, such as cognitive behavioral therapy (CBT), mindfulness-based interventions (MBIs), interpersonal psychotherapy, and social support therapy, are commonly applied in the treatment of SUD with limited long-term effectiveness. For example, we and other researchers found that mobile-based CBT can improve quitting smoking rate in the early stage, but most of quitters eventually relapsed (Liao *et al*., 2018b).

Here, we review recent advances in neuroimaging studies underlying the addictive behaviors and brain circuits related to the three stages of addiction, and expect to provide considerable insight into the neurobiological mechanisms of SUD. We also focus on the related treatment and promising interventions.

### Diagnostic criteria, definition, and conceptual framework of SUD

From the perspective of diagnostic concepts, SUD is a broader concept that encompasses the term of drug addiction. Since 2013, SUD has appeared in DSM-5, with the combination of two separate conceptions used previously (substance abuse and substance dependence) (Hasin *et al*., 2013). According to the DSM-5, SUD is chronic relapsing neuropsychiatric disorder characterized by three main features: (i) compulsive seeking and taking of drugs, (ii) loss of control and craving in limiting intake, and (iii) emergence of a negative emotion states (e.g. dysphoria, anxiety, and irritability) and stress. Over 10 types of addictive substance can lead to SUD, with alcohol, tobacco, caffeine, cannabis, methamphetamine, heroin, and cocaine as most common ones (Vahia, 2013).

SUD has been associated with severe economic and social consequences (e.g. illness, death, low productivity, and crime). The number of cases of SUD reached an estimated 71.2 million people globally in 2017 (Pan *et al*., 2020). According to the United Nations Office on Drugs and Crime (UNODC), an estimated 192 million people aged 15–64 recreationally used marijuana in 2018. In the USA, 21.6 million people over the age of 12 meet the criteria for SUD (Baysinger and Gianessi, 2015). In China, mental, neurological, and substance use disorders together contribute to nearly 80% disease burden of years lived with disability (Charlson *et al*., 2016). For alcohol and tobacco, most people in America, Europe, Japan, and New Zealand reported lifetime use of them, with 74% in US, 67% in Lebanon, and 60% in Mexico (Degenhardt *et al*., 2008). In China, male, female and total drinking rates were 84.1, 29.3, and 59.5%, respectively, and 6-month incidence rates of acute intoxication were 5.162, 0.017, and 2.637% (Wei *et al*., 1999). In Beijing and Shanghai, alcohol abuse (DSM-IV) rate was 4.7% in 2001 (Lee *et al*., 2007). As for heroin, methamphetamine, ketamine, and other illicit drugs, over 14 million addicts in China as indicated in the 2021 China Drug Situation Report.

The addiction progress is a cycle that involves transformation from positive reinforcement (driven by impulsivity) to negative reinforcement (driven by compulsivity). The conceptual spiraling distress–addiction cycle was first published in *Science* in 1997 by George F. Koob who provided a theoretical framework for understanding the neurobiological mechanisms of addiction (Koob and Le Moal, 1997). Since then, the conceptual framework of SUD has been proposed in a series of reviews by Koob (Koob and Volkow, 2010, 2016; Koob, 2021). This composite addiction cycle contains three major components—binge/intoxication, withdrawal/negative affect, and preoccupation/anticipation—that correspond to three domains of dysfunction (incentive salience/pathologic habits, negative emotional states, and executive function, respectively) (Koob, 2021). Interactions and neuroplasticity changes of these three stages make the dysfunction more intense and ultimately become addiction.

### Overview of neuroimaging techniques for SUD

Over the past two decades, our knowledge of the neurobiological mechanisms of SUD is largely thanks to two predominant developments. One is the neurocircuit findings on multiple animal laboratory models of addiction. Another is the unprecedented advances in neuroimaging technologies, and studies of the human brain using these noninvasive techniques at both molecular and neurobiological levels (Koob and Volkow, 2010). These imaging tools can be vital for diagnosis, treatment monitoring, and finding prediction biomarkers in neurologic and psychogenic diseases (Risacher and Saykin, 2021). The current neuroimaging used clinically encompasses mainly three types: positron emission tomography, electroencephalography (EEG), and magnetic resonance imaging (MRI). Owing to its advantages (safety and flexibility in the information obtainment), MRI is most frequently used and has become the mainstay in SUD imaging studies (Suckling and Nestor, 2017).

Positron emission tomography, using radioactive compounds, can be used *in vivo* to radiolabel and detect the neurotransmitters (e.g. DA and DA receptors) binding to the drugs or ligands of substances in the brain (Wagner *et al*., 1984; Brody *et al*., 2004; Scott *et al*., 2007). The pharmacokinetics and distribution of drugs or metabolites can also be detected (Volkow *et al*., 1991; Volkow *et al*., 1997a; Volkow *et al*., 1997b). EEG is another physical recording of voltage between two different cerebral plots reflecting the summation of postsynaptic potentials from cortical neurons. Using EEG, we can observe both local and global cortico-cortical circuitry underlying SUD (Thatcher *et al*., 1986; Alper *et al*., 1990) or even predict relapse in patients with SUD (Winterer *et al*., 1998).

MRI emerged in the 1970s (Blamire, 2008). Using a magnet and radiofrequency energy, MRI visualizes the internal structure and soft tissue morphology of human body, especially for imaging the brain (Zhu *et al*., 2015). Generally, MRI draws brain structural changes via voxel-based morphometry for measuring grey matter volumes, diffusion tensor imaging for white matter tracts, and draws brain functional changes via blood oxygenation level-dependent endogenous contrast for detecting local function of specific regions and circuits (Ashburner and Friston, 2000, 2001; Biswal, 2012; Suckling and Nestor, 2017; Yousaf *et al*., 2018). In addition, relaxation times characterized two different time constants, T1 and T2, according to the texture of tissues (solid tissues such as gray and white matter have shorter relaxation times than fluids such as water and cerebrospinal fluids) (von Schulthess *et al*., 2013).

Taken together, neuroimaging tools give us a chance to get closer to the brain molecular and circuit mechanisms of SUD. The studies of animal models and subsequently brain imaging studies on addicted individuals at different stages of addiction have enhanced our understanding of SUD, and provide insights into preventing and treating this disorder.

### Neurobiological mechanisms and brain circuits of SUD

#### Binge/intoxication stage

Intoxication is the first stage of the addiction cycle when individuals are exposed to an overdose of drugs, which produces significant behavioral and cognitive impairments. These compulsive behaviors are considered critical for the transition from casual uptake to addictive substance use and are influenced by positive reinforcing stimuli (such as a euphoric effect) (Volkow *et al*., 1997b; Adinoff, 2004). This process is now well understood and is mainly involved in reward systems and DA motive system (Wise, 2004; Volkow *et al*., 2017). Evidence from single drug administration has shown that the mesolimbic DA system is activated by acute exposure to opioids, ethanol, nicotine, amphetamine, and cocaine (Di Chiara and Imperato, 1988). Traditional neuroimaging studies have revealed that increased extracellular DA in the mesolimbic system, especially the nucleus accumbens (NAc), is associated with drug exposure or the motivational characteristic stimuli such as drugs (Table 1) (McGregor and Roberts, 1993; Robledo and Koob, 1993; Volkow *et al*., 1997b; Salamone *et al*., 2007). DA neurotransmission in NAc was detected increased in response to cues related to cocaine (Kawahara *et al*., 2021). However, the latter findings pointed out that activation of NAc was not enough for acute reinforcing effects (brain reward systems seem to have a multidimensional neuropharmacological basis, see a review in Koob, 1992) as 6-hydroxydopamine failed to block heroin or ethanol self-administration (Hnasko *et al*., 2005; Nestler, 2005). In addition, Nora Volkow *et al*. found that cocaine worked by blocking the DA transporter and thereby increased the free concentration of DA in the brain (Volkow *et al*., 1997b).

**Table 1:** Brain regions and neurotransmitter systems involved in the three stages of addiction.

Functional domain	Main system involved	Brain region	Neurotransmitter system
binge/intoxication stage	reward circuit of the midbrain limbic system	NAc	dapamine (McGregor and Roberts, 1993; Salamone *et al*., 2007)
		VTA	dapamine, opioid (Mameli-Engvall *et al*., 2006; Matsumoto and Hikosaka, 2009)
		PFC	glutamate (Wu *et al*., 2018)
		CN	dapamine, seroton (Kuczenski *et al*., 1995)
		CeA	dapamine, serotonin (Koob, 1999)
withdrawal/negative affect	anti-reward/pressure circuit	PFC	dapamine (Cassens *et al*., 1981; Barr *et al*., 1999)
		OFC	serotonin (Wright *et al*., 2021)
		ACC	dapamine (McDevitt *et al*., 2021)
		BNST	norepinephrine, catecholamine (Park *et al*., 2012), κ-opioid (Lê *et al*., 2018)
preoccupation/anticipation	executive control or inhibitory control	PFC	glutamate, dapamine (Parsegian and See, 2014)
		BNST	norepinephrine (Shaham *et al*., 2003)
		Insula	corticotropin-releasing factor (Naqvi and Bechara, 2009)
		LDT	acetylcholine, glutamate, γ-aminobutyric acid (Mantsch *et al*., 2016; Kaneda, 2019)

LDT = the laterodorsal tegmental nucleus.

Reward systems and goal-directed behaviours also involve other brain regions such as the VTA. Animal studies have shown opioids and alcohol can be directly self-administered into the VTA and intravenous nicotine self-administration is blocked by neurotoxin-specific lesions of the DA system (Watkins *et al*., 2000). Putative VTA DA neurons are activated in response to both unexpected and expected rewards (Matsumoto and Hikosaka, 2009; Eshel *et al*., 2016), and recent optogenetic studies indicate that activation of the VTA DA neurons is rewarding (Witten *et al*., 2011). Further detailed study reveals two types of DA neuron in the VTA delivering positive and negative motivational signals (Matsumoto and Hikosaka, 2009). Moreover, nicotine is reported to elicit DA release by activating beta2 subunit of acetylcholine receptors in the VTA (Mameli-Engvall *et al*., 2006) and also accelerates endogenous opioid release, which contributes to rewarding effects and synaptic function (Table 1) (Mansvelder and McGehee, 2000). These results have illustrated that the VTA DA neurons play an important role in the reward system in the intoxication stage.

Along with mesolimbic areas in DA reward system, other regions such as the PFC, caudate nucleus (CN) area, and central nucleus of the amygdala (CeA) are also considered to have a key function in the acute reinforcement (Table 1). MRI studies found that the PFC not only regulated limbic reward regions but was also activated with exposure to cocaine-related cues (Goldstein and Volkow, 2011; Kawahara *et al*., 2021). Our magnetic resonance spectroscopy results found metabolites (glutamine) of medial PFC significantly increased in methamphetamine addicts compared with healthy control participants (Wu *et al*., 2018). A study also confirmed the PFC in modulation of the VTA DA neurons is associated with nicotine reward and the PFC-VTA functional coupling is one mechanism for nicotine addiction (Wu *et al*., 2013). Electrophysiological recording of neurons in the CN showed both acute and chronic methylphenidate exposure result in motivational changes (Venkataraman *et al*., 2020). Functional MRI (fMRI) analyses also revealed the CN activation in smokers (Qian *et al*., 2017). The CeA is another key area in reward. Neurochemical elements such as DA and serotonin increase in the CeA lead to compulsive drug-seeking (Table 1) (Koob, 1999). Optogenetic activation of the CeA generates addiction-like behavior related to reward and adverse consequences (Tom *et al*., 2019).

#### Withdrawal/negative affect

Chronic drug exposure results in adaptive changes of brain receptors and neurotransmitters and relative circuits. The anti-reward/pressure circuit activated when drug administration is suddenly interrupted, and neurotransmitters such as DA and 5-HT in this circuits also decrease (Koob and Volkow, 2016). In the withdrawal/negative affect stage, individuals with SUD show varieties of negative emotional symptoms including irritability, malaise, dysphoria, anhedonia, and state of stress, which are involved in negative reinforcement associated with brain stress system consisting of the PFC, ventral striatum, and EA (Cassens *et al*., 1981; Gawin and Kleber, 1986; Koob *et al*., 2014). The emergence of this negative emotional state might be due to the adaptive response and decreased sensitivity to the repeated DA stimuli (Table 1) (Cassens *et al*., 1981; Barr *et al*., 1999). The EA is a forebrain area that consists of the bed nucleus of the stria terminalis (BNST), CeA, and possibly a transition zone in the medial portion (or shell) of the NAc (Heimer and Alheid, 1991).

The EA is the most relatively region in antireward circuit with tolerance and withdrawal behaviors in the negative effect stage (Koob, 1999; Baidoo and Leri, 2022). Chronic exposure to alcohol changes the synaptic function and neuronal excitability both in the prefrontal cortical and EA regions (Pleil *et al*., 2015). Neuronal activity-regulated pentraxin expressed in the EA is found to play a central role in opioid withdrawal (Reti and Baraban, 2003). More recently, a study demonstrates the VTA DA projection to the amygdala (VTA^DA^→amygdala) is necessary and sufficient for reinstatement of cocaine place preference (Tian *et al*., 2022).

The PFC, including the orbitofrontal cortex (OFC) and anterior cingulate cortex (ACC), is activated in the intoxication/bingeing stage and deactivated in withdrawal stage (Goldstein and Volkow, 2002). One recent study in rats illustrated Fos expression (marker for neuronal activity) in the OFC increased under incubation of craving and the OFC inactivation decreased drug seeking on withdrawal day 15, which indicated that the OFC plays a critical role in craving and withdrawal (Altshuler *et al*., 2021). On the other hand, the OFC serotonin (5-HT) function of pyramidal neurons and 5-HT1A and 5-HT2A receptor were reported to mediate cocaine withdrawal behaviours (Table 1) (Wright *et al*., 2021). Most recently, the activity and D1-receptor of layer V pyramidal neurons in the ACC (ACC L5 PyNs) was shown to play a central role in opioid-induced withdrawal and withdrawal-induced mechanical hypersensitivity (McDevitt *et al*., 2021). However, glutamate concentration in the ACC and cingulate-cortical functional connectivity is found to be participated in nicotine deprivation (Abulseoud *et al*., 2020).

The increases in stress and anxiety-like behaviors in the withdrawal stage correlate with BNST functions connecting stress and reward neural circuitry. Norepinephrine or catecholamine (Table 1) (Park *et al*., 2012) in the BNST and noradrenergic receptors modulating excitatory and inhibitory transmission in BNST is influenced by withdrawal related stress (Flavin and Winder, 2013). Moreover, κ-opioid receptors in BNST is involved in stress-related alcohol seeking as site-specific injections of the κ-opioid antagonist induced Fos expression with reinstatement indicted (Table 1) (Lê *et al*., 2018). On the other hand, *ex vivo* slice physiology shows that increased neuronal excitability in the BNST is associated with ethanol-induced conditional place preference (Pati *et al*., 2019).

#### Preoccupation/anticipation (craving)

Craving refers to the subjective experience of the impulse or desire to use addictive substances, with no significant difference between the female and male individuals (Nicolas *et al*., 2022). As a key clinical feature in SUD, craving has been re-incorporated into the diagnostic criteria of DSM-5 and hypothesized to be a key element in addiction and relapse.

Craving may involve changes in multiple neural circuits and brain regions related to the core symptoms of addiction such as negative emotion, impulse, or compulsive motivation. Executive control or inhibitory control is vital for maintaining goal-directed behaviour and is thought to be a key factor in craving and relapse (Li and Sinha, 2008). Substantial evidence summarizes that the prefrontal-limbic dysfunction contributes to attenuation of inhibitory control in SUD (Menon and D'Esposito, 2022) and involves in the anticipation stage. One animal study suggests that brain nuclei such as the dorsal PFC, core of the NAc (NAcore), ventral pallidum, and dorsal PFC–NAcore–ventral pallidum circuit mediates cocaine-induced reinstatement. Moreover, DA administration into the PFC can elicit a reinstatement in cocaine self-administration model (McFarland and Kalivas, 2001).

In addition, cue-induced reinstatement involves a glutamatergic regulation and projection in the PFC (Table 1) (Parsegian and See, 2014), insular cortex (Zhang *et al*., 2019), and prelimbic cortex (McGlinchey *et al*., 2016; Stefanik *et al*., 2016) in multiple SUDs, while stress-induced reinstatement is associated with D1/D2 DA receptor in the limbic and motor circuitry (McFarland *et al*., 2004) or cholinergic, glutamatergic, and gamma-aminobutyric acid (GABA)-ergic projection in the laterodorsal tegmental nucleus (Kaneda, 2019). Futhermore, neuropharmacological studies show the involvement of corticotropin-releasing factor and noradrenaline in stress-induced reinstatement (Mantsch *et al*., 2016).

Structural and functional MRI studies have found that a variety of brain regions are impaired under addiction while abstinence can rescue these brain damages. Using diffusion tensor imaging, our laboratory and others provide evidence for white matter abnormalities following chronic ketamine exposure both in monkeys (Li *et al*., 2017) and ketamine users (Liao *et al*., 2010). We found that white matter in the bilateral frontal and left temporoparietal regions changes in chronic ketamine abusers in a dose-dependent manner indicating a microstructural basis for the behavior changes with prolonged ketamine use. Meanwhile, we also observed reduced gray matter volume in the dorsal prefrontal regions (the left superior frontal gyrus and right middle frontal gyrus) in chronic ketamine users. Indeed, abstinence and effective relief of craving is expected to ameliorate brain damage caused by addictive substances. For example, He *et al*. found that white matter damage in the DLPFC could be improved in short- or long-term cocaine abstinence patients over 5 years (He *et al*., 2020). Our study found that the gray matter density of the PFC, ACC, and temporal cortex decreased after 3 days of abstinence, and the damage of the superior frontal gyrus reversed after 1 month of withdrawal, but there were no changes in the right middle frontal gyrus, left cingulate gyrus, and inferior occipital gyrus (Wang *et al*., 2012). Chen *et al*. found that heroin abstinent patients had lower levels of evoked craving, stronger functional connectivities between the dorsal ACC, left DLPFC, and right posterior parietal cortex, which are positively correlated with duration of abstinence (Chen *et al*., 2021). In addition, fMRI revealed the increase of regional homogeneity (ReHo, first reported by Zang *et al*., 2004) of the left precentral frontal gyrus and the decrease in the right ACC were related to the decreased craving (Liao *et al*., 2012).

Other nuclei were also sporadically reported to be associated with drug anticipation. One study shows increased craving after abstinence is associated with decreased amygdala volume in alcohol abusers (Wrase *et al*., 2008). Our previous study found that the degree of caving in ketamine addicts is proportional to the amount of ketamine use and the functional connectivity between the posterior parietal lobe and the right dorsal nucleus was also related to craving (Liao *et al*., 2016). Imaging studies found that cannabis, cocaine, and heroin users all showed abnormal activation in the anterior insula, DLPFC, and inferior parietal lobe compared with healthy control participants using a cue-induced craving task (Zilverstand *et al*., 2018). One study found ketamine addicts exhibited increased activation in the anterior cingulate gyrus and precuneus than healthy control when exposed to ketamine-related clips, while the anterior central frontal gyrus was mainly activated under smoking-related cues (Liao *et al*., 2018a).

### Current clinical treatments of SUD

The past 300 years have seen the great advances in neurobiological mechanisms in SUD. Ongoing studies not only broaden our knowledge of the molecular pathways and brain circuits involved in SUD, but also provide new ideas and targets for addiction treatment. Overall, the current therapeutic options in clinical practice for SUD are still far from satisfactory (Angres and Bettinardi-Angres, 2008; Liu and Li, 2018). The FDA approved medications including buprenorphine, naloxone, methadone, acamprosate, and some kinds of physical therapy for SUD have a limited effectiveness and insufferable side-effects (Volkow and Boyle, 2018). As SUD is a chronic brain disease with genetic, molecular, environmental, and psychosocial factors involved, it is necessary to figure out the key pathogenic factors beneficial to the treatment. Here, we briefly review main clinical treatments for SUD, including pharmacological, physical (brain stimulation), and psychological therapies (Leshner, 1997).

### Pharmacological therapies

The present pharmacological treatments for SUD mainly target to neurotransmitters and neural networks in process of SUD (e.g. the reward system, the antireward system) (Liu and Li, 2018). For example, buprenorphine/naloxone or a combination medication of buprenorphine and naloxone is an opioid agonist used to treat opioid use disorder and chronic pain. It works typically by relieving withdrawal and craving symptoms (Khroyan *et al*., 2015). Acamprosate, the medication treatment used in alcohol use disorder, is believed to act as an NMDA receptor antagonist and modulator of GABA_A_ receptors (Liang and Olsen, 2014). Medications for nicotine use disorders approved by FDA are nicotine receptor antagonist bupropion (Rigotti *et al*., 2022), partial agonist varenicline (Ebbert *et al*., 2015), and nicotine replacement therapy (Molyneux, 2004). However, the current medications for SUD failed to prevent a high relapse rate, and some of them even show serious side-effects (George and O'Malley, 2004).

### Physical therapies

Brain stimulations targeting specific regions involved in the cycle of addiction have been proved effective in reducing drug use and relapse. The most frequently used and studied among noninvasive brain modulations are transcranial magnetic stimulation (TMS) and transcranial direct current stimulation (tDCS) (Lupi *et al*., 2017; Hanlon *et al*., 2018). Other neuromodulatory treatment for addiction also includes deep brain stimulation (DBS), which directly stimulates and modulates the brain (Luigjes *et al*., 2019).

TMS, particularly repetitive TMS (rTMS), has been approved by FDA to treat depression. Recently, it has been found that TMS may also be effective in drug addiction treatment, and has certain effects in various SUDs (such as cannabis, cocaine, and opioid use disorders) (Stein *et al*., 2019; Steele and Maxwell, 2021; Nardone *et al*., 2022). Studies have shown that both rTMS and tDCS targeting the DLPFC is curative to reduce craving and prevent relapse (Fregni *et al*., 2008; Coles *et al*., 2018). For example, Yuan and colleagues found that in relative to sham groups, 10 consecutive days of 1 Hz rTMS over the left DLPFC can be effective in reducing craving scores and impulsive behavior in heroine abusers (Yuan *et al*., 2020). Li *et al*. demonstrated that, compared to the wait list control group, either 20 daily consecutive sessions of high frequency (10 Hz) or low frequency (1 Hz) rTMS can decrease craving scores in heroin addicts with treatment effects up to 60 days (Liu *et al*., 2020). High-frequency rTMS in the left DLPFC can also improve cognitive abilities in individuals with SUD and even normal people, including working memory, attention, and learning ability (Gorelick *et al*., 2014; Wang *et al*., 2014) Although accumulated evidences from randomized controlled trials (RCT) have revealed the short-term efficacy of TMS in patients with SUD, the long-term outcome efficacy, such as 6 or 12 months, and the underlying mechanisms remain unknown.

The tDCS treatment for SUD also shows promising results. An RCT demonstrated that tDCS with the cathode over the left DLPFC and the anode over the right DLPFC (2 mA, 20 min, 5 days) can decrease craving and anxiety scores compared to the sham-tDCS group (Batista *et al*., 2015). Using bilateral tDCS (left cathodal and right anodal) targeted over the DLPFC (2 mA, 13 min, 5 days), a study found significant decrease in relapse rate at 6 months. However, most studies were based on small samples, well-designed large-sample size RCTs are needed to verify its efficacy for preventing relapse of SUD.

Since DBS has shown effective in numerous animal studies for addiction to various drugs of abuse (Creed *et al*., 2015; Guercio *et al*., 2015; Hamilton *et al*., 2015; Batra *et al*., 2017), recently, several case series or case reports in humans have presented promising results. NAc, a key structure in the mesolimbic reward pathway, is the most common used target in DBS. In the case report by Müller *et al*. (Müller *et al*., 2016), five patients who received DBS of the NAc to treat their treatment-resistant alcohol addiction all had encouraging results. In another study (Kuhn *et al*., 2011), the patients showed a reduction of alcohol craving and intake 1 year after treatment. Other studies examined the effect of bilateral high-frequency NAc stimulation in patients with a chronic treatment resistant course of heroin or cocaine dependence, and also reported promising effects (Kuhn *et al*., 2014; Gonçalves-Ferreira *et al*., 2016). It should be noted that more RCTs are needed to verify efficacy and for long-term abstinence and potential synergy with other addiction interventions in the future.

### Psychological therapies

In the recurring cycle-binge/intoxication stage, the core of treatment is to increase patients’ motivation to quit, in which case motivational interviewing (MI) might be more useful. MI is a client-centered therapeutic intervention that aims to solve ambivalence toward change (Hettema *et al*., 2005). A review showed that MI reduced the use of substances compared to control group (Smedslund *et al*., 2011). A meta-analysis showed the brief MI also performed well in reducing alcohol consumption, but a single MI is less effective than a combination of MI with CBT (Riper *et al*., 2014).

During the second stage, the most important thing is to relieve withdrawal symptoms, such as dealing with various negative emotions and reducing negative urgency . Thus, improving emotion regulation seems useful in this period. CBT can enhance adaptive emotion regulation skills, and help individuals perceive emotions and improve personal ability to adjust to these emotions (Berking *et al*., 2008). A study reported that CBT could alleviate negative urgency in people with gambling disorder (Garcia-Caballero *et al*., 2018). Mindfulness meditation is often described as nonjudgmental attention or regulation according to present experiences. An RCT indicated that MBIs can reduce drug use (Tang *et al*., 2016). Eric L. Garland found that MBIs could synergize a range of positive affective mechanisms to reduce addictive behavior (Garland, 2021).

In the stage of preoccupation/anticipation (craving), the key to relapse is the craving for drugs, as most withdrawal symptoms will subside in a short period of time except for craving. Hence, the core of treatment in this stage includes cope with craving and preventing relapse. A systematic review showed CBT has efficacy in reducing the use of methamphetamine and deceasing craving (AshaRani *et al*., 2020). Our large-sample size RCT revealed the 24-week cessation rate in the CBT-based smoking cessation intervention group was 3-fold higher than that in the control group (Liao *et al*., 2018b). A line of study examined the efficacy of MBIs for SUD, and one RCT indicates a better long-term efficacy of MBIs than CBT (Bowen *et al*., 2014). Mindfulness strategies could bring about immediate reductions in craving, which suggests these strategies may confer unique benefits of both reducing craving and decreasing the extent to which craving leads to drug use (Tapper, 2018).

## Conclusions and future directions

SUD has been characterized as a chronic, relapsing brain disorder with complex etiology and pathogenesis. In this review, we summarize neurobiological mechanisms, neurotransmitters, and brain circuits involved in the three-stage cycle conceptual framework of SUD, mainly focusing on the neuroimaging findings. Imaging studies provide us a chance to closely explore the global changes in the brain areas related to previous well-known reward system, antireward/antistress system, and further explore the underlying neural mechanisms of craving in the process of addiction. Accordingly, based on these findings, pharmacological, physical, and psychological treatments for SUD are included. Clearly, however, a great deal of evidence and advances are observed in SUD, the current treatments of SUD are unsatisfactory, leaving us a long way to go.

We emphasize that strong craving after the drug withdrawal as an important target in the treatment of SUD. Thus, both physical therapies and CBT targeted to the DLPFC regulating craving show certain therapeutic effects. However, traditional CBT is mainly focused on content and little attentioni s paid to the change and internal needs of different individuals in the process of the addiction cycle, which may be the reason for the less satisfactory treatment effect. Clinical psychological intervention based on the core characteristics of the different stages of addiction is expected to achieve therapeutic breakthroughs, and the analysis of the neurobiological mechanisms related to therapeutic effects is conducive to finding better treatment methods (Volkow *et al*., 2016). The key to improve treatment effects is to target the core symptoms, and meet the internal needs of individuals at any of these three stages, which may more effectively relieve drug craving and reduce relapse.

The physical therapies, such as rTMS and tDCS, were commonly used for their noninvasive features. Besides the stimulation parameters and duration of treatment, an optimal therapeutic target is a puzzle and a key to the efficacy of TMS. Early TMS trials positioned the TMS coil over the PFC using scalp measurements. Using the “5–6 cm” method and the later “10–20 electroencephalography system”, many larger clinical investigations have been approved by the FDA (see a review by Cash *et al*., 2021). To target the superficial cortex is easy, but, in most cases, the regions involved in addictive characteristics may be located deep in the brain. The cross-synaptic connection localization method has emerged. Using functional or structural connectivity methods to locate the superficial cortical brain region corresponding to the hub region in the neural circuit, this method achieves the similar effect of direct regulation of the deep brain in the core of the neural circuit (Drysdale *et al*., 2017; Siddiqi *et al*., 2022). A neuronavigational approach was used according to differences in brain structure, function, and metabolism of individuals with depression (Herwig *et al*., 2003; Fitzgerald *et al*., 2009). Personalizing the stimulation site based on individual connectivity has been reported to yield very high response and remission rates. One study has increased working memory in healthy individuals by targeting the cortical-hippocampal network using personal functional connectivity rTMS under MRI guidance (Wang *et al*., 2014). To improve the current unsatisfactory efficacy of TMS or tDCS, the following main factors as well as challenges involved in rTMS therapy should be addressed: (i) targeting to a specific brain region or neural circuits corresponding to addiction behaviors; (ii) targeting the regions in the deep brain; and (iii) finding individualized brain regions based on functional connectivity and network with MRI.

In summary, neuroimaging techniques advanced our understanding of neurological mechanisms of SUD. Multiple brain regions and circuits are involved in the addition cycles and are likely to contribute differentially to the complex behaviors in different individuals with SUD. The current therapies of SUD are limited, and relapse remains a major clinical challenge. Therapies targeting the characteristics of the SUD at these different stages, and meeting the dynamically changed needs of individuals with SUD throughout the three stages of addiction cycle, may be the direction of future treatment for SUD.
